# Review of the genus *Hypocala* Guenée (Lepidoptera, Erebidae, Hypocalinae) from Chinese fauna

**DOI:** 10.3897/BDJ.14.e217960

**Published:** 2026-10-29

**Authors:** Asad Bashir, Zhaofu Yang

**Affiliations:** 1 Laboratory of Plant Protection Resources and Pest Management, Ministry of Education, Entomological Museum, College of Plant Protection, Northwest A&F University, Yangling, 712100, China Laboratory of Plant Protection Resources and Pest Management, Ministry of Education, Entomological Museum, College of Plant Protection, Northwest A&F University Yangling, 712100 China

**Keywords:** checklist, distribution, genitalia, host plants, identification key, morphology, taxonomy

## Abstract

**Background:**

The genus *Hypocala* Guenée, 1852 (Lepidoptera: Erebidae, Hypocalinae) was previously treated in the *Fauna Sinica* volume on Noctuidae, which recognised four species, based on external morphology from China: *H. subsatura* Guenée, 1852, *H. deflorata* (Fabricius, 1794) *H. rostrata* (Fabricius, 1794) and *H. violacea* Butler, 1879. A fifth species, *H. andamana* Wileman, 1923 is known from Taiwan, based on literature records, but was not available for examination in this study.

**New information:**

In the present study, based on examination of available museum material, we provide updated diagnoses, descriptions and illustrations of adults and male genitalia for all four species, as well as female genitalia for two species where material was available. A taxonomic key to the Chinese species, updated synonymies and host plant records are also provided. This study establishes a comprehensive baseline for future taxonomic and pest-management research on *Hypocala* in China.

## Introduction

The subfamily Hypocalinae Guenée, 1852 is one of the smallest lineages within the Erebidae, currently comprised of an estimated 31 species in five genera (Sisson 2025). Historically, the classification of this group has been unstable. It has long been treated as a tribe (Hypocalini) within the Noctuidae, but Lafontaine and Schmidt (2010) recommended elevating the group to subfamily status, based on characters of the genitalia and mitochondrial cytochrome c oxidase subunit I (*COI)* sequence data that appeared to unite the group into a monophyletic assemblage. This action was subsequently supported by Zahiri (2012), whose molecular phylogenetic analysis of Erebidae recovered the Hypocalinae as a monophyletic group closely related to the Eulepidotinae, albeit with weak support for that sister relationship. Following these works, Hypocalini was officially elevated to Hypocalinae within the Erebidae (Zahiri 2012). The generic composition of Hypocalinae has also been contentious. For example, the genus *Aon* has traditionally been placed in several different subfamilies across both the Noctuidae and Erebidae. However, both *COI* and morphological characters have since united *Aon* with the other genera of Hypocalinae (Lafontaine and Schmidt 2010), providing a more stable generic framework for the subfamily.

Within Hypocalinae, the genus *Hypocala* Guenée, is the most widespread and economically significant lineage with its species distributed primarily in the Old-World tropics and the greatest diversity in South and Southeast Asia. The genus is of agricultural importance because the larvae feed on *Diospyros* (Ebenaceae) and other fruit trees and several species are considered pests of persimmon and apple tree.

In China, the genus was last comprehensively treated in the *Fauna Sinica* volume on Noctuidae (Chen 1999), which recognised four species: *H. subsatura* Guenée, *H. deflorata* (Fabricius), *H. rostrata* (Fabricius) and *H. violacea* Butler. However, genitalic features were missing in that book and from subsequent regional treatments (Li 2023; Xian 2024). Five species of *Hypocala* have since been recorded from China, including *H. andamana* Wileman, 1923 from Taiwan (Holloway 2005); however, no specimens of this species were available for examination in this study. In this study, we review all four Chinese species and provide detailed descriptions, including male and female genitalia, based on examinations on available museum material. *H. violacea* from Hainan is also compared with material from other regions. In addition to the mainland Chinese species treated here, we also incorporate literature records for species occurring in Taiwan, Macao and Hong Kong to provide a comprehensive overview of the genus *Hypocala* in the Chinese fauna (Holloway 2005, Li 2023).

## Materials and methods

**Identification and specimen sources**

Species were identified, based on external morphological characters including wing venation, wing pattern (fore-wing shape, apex and colour; hind-wing colouration) and head features (development of the proboscis, frons and labial palpi) and genitalic structures, primarily following the taxonomic keys and descriptions provided by Chen (1999) and Holloway (2005). Specimens examined in this study were obtained from the Entomological Museum of Northwest A&F University, Yangling, China.

**Genitalic dissection**

Male and female genitalia were dissected following standard procedures (Landry 2007; Yang 2012). The abdomen was macerated in 10% potassium hydroxide (KOH) overnight, rinsed in distilled water and examined under a stereomicroscope. The dissected genitalia were dehydrated through a graded ethanol series (50%, 75%, 85%, 95% and 100%), mounted on a microscope slide in Canada balsam and covered with a coverslip. Each slide was assigned a unique preparation number and labelled with the corresponding specimen information.

**Imaging and figure preparation**

Adult specimens were photographed using a Canon digital camera. Images of the genitalia were obtained with a Zeiss Stereo Discovery V20 stereomicroscope (Carl Zeiss, Oberkochen, Germany). Image processing and figure preparation were carried out in Adobe Photoshop 2023 v.24.1.1 (Adobe Systems Inc., San Jose, California, USA), with adjustments restricted to brightness, contrast and cropping.

## Data resources

The primary data for this study are the specimens examined from the Entomological Museum of Northwest A&F University (NWAFU), representing four *Hypocala* species (*H. subsatura*, *H. violacea*, *H. deflorata* and *H. rostrata*). Genitalic dissections were prepared and deposited in the NWAFU collection. All examined specimens are deposited at NWAFU and are publicly accessible for future study. Distribution records for Taiwan, Macao and Hong Kong are based on published literature (Holloway 2005; Li 2023) as no specimens from these regions were available for examination in the present study.

## Taxon treatments

### Hypocala

Guenée, 1852

A988B114-5572-5E73-B80F-E8753514B1FE

Hyblaea deflorata Fabricius, 1794.

#### Diagnosis

Proboscis well developed. Frons flat and smooth, with a tuft of hair above. Labial palps extending forwards, the third segment angled downwards, densely scaled, forming a triangle. Male antennae with short setae. Thoracic scales smooth. Tibiae without spines, covered with sparse hairs. Fore-wing relatively narrow, apex slightly rounded, outer margin nearly perpendicular to costa, colouration highly variable. Hind-wing dark brown with orange-yellow markings. Larvae with four pairs of abdominal prolegs, fully developed.

#### Distribution

China; Russia, Japan, North Korea, South Korea; Myanmar, India, Nepal, Bangladesh, Sri Lanka, Vietnam, Thailand, Malaysia, Indonesia, the Philippines, Papua New Guinea; USA (Hawaii), Africa.

### Hypocala subsatura

Guenée, 1852

31157B8D-7244-5671-A4D2-3D27851519A0

Hypocala subsatura Guenée (1852): 75. Type locality: Silhet [Sylhet, Bangladesh].
Hypocala aspersa Butler (1883): 164.
*Hypocala subsatura var. limbata* Butler (1889): 76.
Hypocala subsatura Warren (1913): 382; Chen (1982): 370; Barlow (1982): 108.

#### Materials

- **Type status:** Other material. **Occurrence:** occurrenceID: 9C43EF1E-C8F0-5AB2-9972-60B3FE80567D; **Material Entity:** materialEntityID: NWAFU:Ento:LepiEreb0077; materialEntityType: Pinned specimen; discipline: Entomology; preparations: pinned | genitalia slide ♂; disposition: in collection; verbatimLabel: CHINA: Shaanxi, Taibai Mountain, Haoping Temple, 1200 m, 13.v.1983 and 14.vii.1983, Shaanxi Taibai Mountain Insect Survey Team
- **Type status:** Other material. **Occurrence:** occurrenceID: A0038E47-4DDD-55E5-A0E0-1C5BC08238FD; **Material Entity:** materialEntityID: NWAFU:Ento:LepiEreb0080; materialEntityType: Pinned specimen; discipline: Entomology; preparations: pinned | genitalia slide ♀; disposition: in collection; verbatimLabel: CHINA: Shaanxi, Taibai Mountain, Luotuoshu, 2100 m, 6.viii.1983, Shaanxi Taibai Mountain Insect Survey Team
- **Type status:** Other material. **Occurrence:** occurrenceID: 107F1F31-32AD-56F0-8F60-5227DAE0AAA0; **Material Entity:** materialEntityID: Pinned specimen; discipline: Entomology; preparations: pinned ♂; disposition: in collection; verbatimLabel: CHINA: Shaanxi, Taibai Mountain, Luotuoshu, 2100 m, 6.viii.1983, Shaanxi Taibai Mountain Insect Survey Team
- **Type status:** Other material. **Occurrence:** occurrenceID: 29FCD8C3-3EC7-586D-A955-2EA3A86A6687; **Material Entity:** materialEntityType: Pinned specimen; discipline: Entomology; preparations: pinned ♂; disposition: in collection; verbatimLabel: CHINA: Shaanxi, Wugong, 3.vii.1981, collected at light trap
- **Type status:** Other material. **Occurrence:** occurrenceID: C785E473-68C6-51BC-B91F-E32AE3729094; **Material Entity:** materialEntityType: Pinned specimen; discipline: Entomology; preparations: pinned ♂; disposition: in collection; verbatimLabel: CHINA: Hainan, Lingshui, Diaoluo Mountain, 2.vi.2007, Wang Yinglun & Li Yankai
- **Type status:** Other material. **Occurrence:** occurrenceID: F86C4E3C-1766-5BC2-AA5B-8F33C1310C44; **Material Entity:** materialEntityType: Pinned specimen; discipline: Entomology; preparations: pinned ♂; disposition: in collection; verbatimLabel: CHINA: Hainan, Lingshui, Diaoluo Mountain, 28.v.2007, Wang Yinglun & Li Yankai
- **Type status:** Other material. **Occurrence:** occurrenceID: BCA47C55-C331-5BCF-A537-DFC03F32F0C6; **Material Entity:** materialEntityType: Pinned specimen; preparations: pinned ♂; disposition: in collection; verbatimLabel: CHINA: Hainan, Jianfengling, 6.vi.2007, Wang Yinglun & Zhai Qing
- **Type status:** Other material. **Occurrence:** occurrenceID: 72EA6086-E48F-5BB8-BE10-3467DC67D929; **Material Entity:** materialEntityType: Pinned specimen; discipline: Entomology; preparations: pinned ♂; disposition: in collection; verbatimLabel: CHINA: Hainan, Jianfengling, 5.vi.2007, Wang Yinglun & Zhai Qing
- **Type status:** Other material. **Occurrence:** occurrenceID: 8244072F-735D-52DB-9734-BEC6FDA4A8FD; **Material Entity:** materialEntityType: Pinned specimen; discipline: Entomology; preparations: pinned ♂; disposition: in collection; verbatimLabel: CHINA: Hainan, Jianfengling, 6.vi.2006, Wang Yinglun & Zhai Qing
- **Type status:** Other material. **Occurrence:** occurrenceID: DF4BC086-B3A0-5AEE-82CC-903802B3A02D; **Material Entity:** materialEntityType: Pinned specimen; discipline: Entomology; preparations: pinned ♂; disposition: in collection ; verbatimLabel: CHINA: Hainan, Wuzhishan, 5.v.2007, Wang Yinglun & Zhai Qing
- **Type status:** Other material. **Occurrence:** occurrenceID: F165FDF5-6E08-5624-B08B-CE0649281402; **Material Entity:** materialEntityType: Pinned specimen; discipline: Entomology; preparations: pinned ♂; disposition: in collection; verbatimLabel: CHINA: Hainan, Baisha, Yinggeling, 22.v.2007, Wang Yinglun & Zhai Qing
- **Type status:** Other material. **Occurrence:** occurrenceID: 6899F956-F2D3-5596-99F9-0AEE5BDE532F; **Material Entity:** materialEntityType: Pinned specimen; discipline: Entomology; preparations: pinned ♂; disposition: in collection; verbatimLabel: CHINA: Yunnan, Jinghong, 23.viii.1987, Zhou Jingruo & Wang Sumei
- **Type status:** Other material. **Occurrence:** occurrenceID: 4744A415-8918-5CAA-ABFB-7DFB598A34E1; **Material Entity:** materialEntityType: Pinned specimen; discipline: Entomology; preparations: pinned ♂; disposition: in collection; verbatimLabel: CHINA: Yunnan, Yiliang, Xiaoshao, 1840 m, 30.vii.2005, collected at light trap
- **Type status:** Other material. **Occurrence:** occurrenceID: 7521B07B-6E9B-5244-83A7-EAD066FDFC87; **Material Entity:** materialEntityType: Pinned specimen; discipline: Entomology; preparations: pinned ♂; disposition: in collection; verbatimLabel: CHINA: Gansu, Wen County, Bifenggou, 20.v.2002, Li Xiushan & Chen Zhenwan

#### Description

**Re-description**

**Figure 1.**
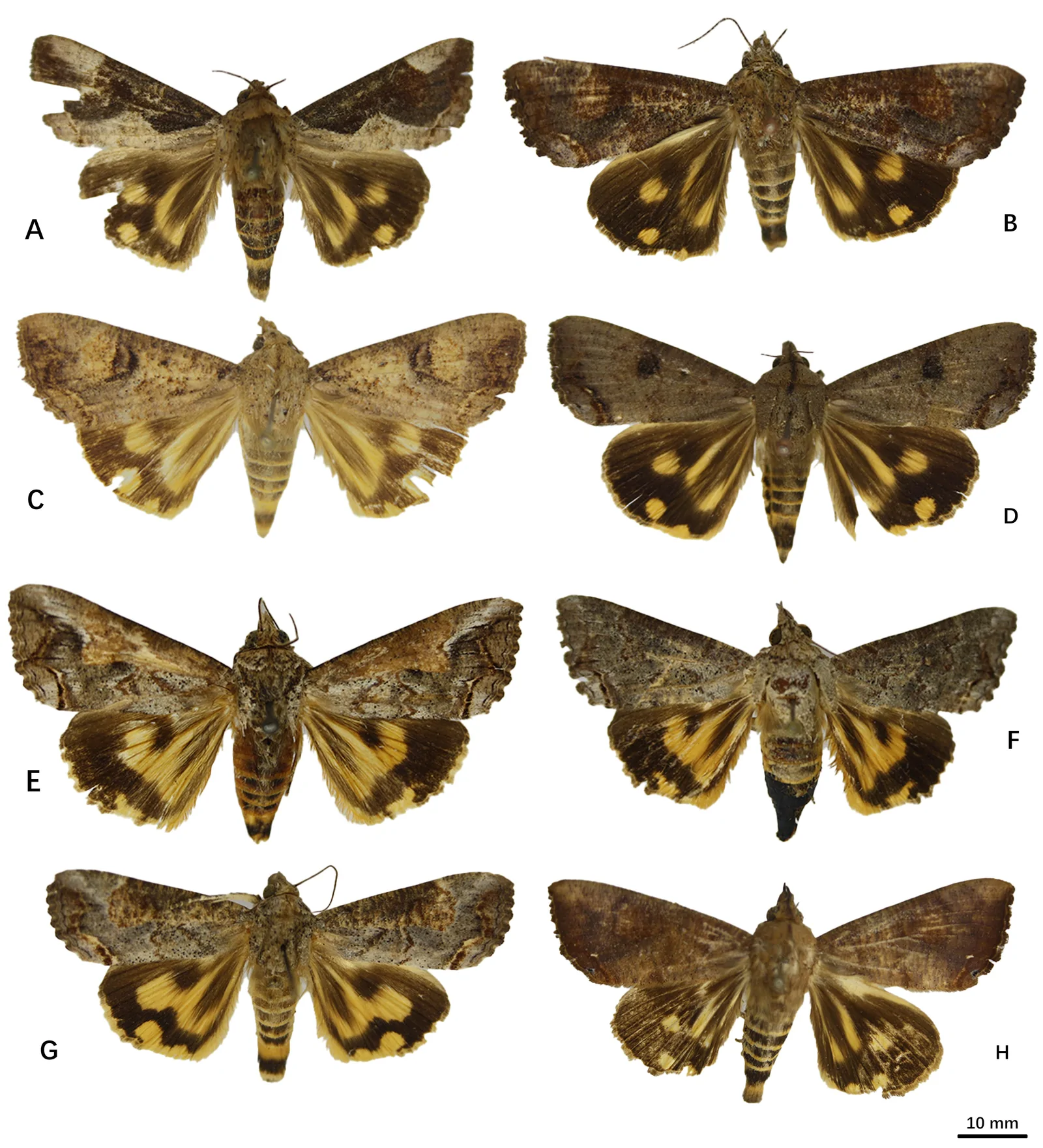
Adults of Chinese *Hypocala* species. **A, B, C** ♂ *H. subsatura*; **D** *H. subsatura* ♀; **E** *H. deflorata* ♂; **F** *H. deflorata* ♀; **G** *H. rostrata* ♂; **H** *H. violacea* ♂.

**Adult (Fig. 1A, B, C** and **D)**. Wingspan 38–42 mm. Head and thorax reddish-brown to dark brown; labial palpi projecting forwards, giving a slightly rostrate appearance. Fore-wing elongate, reddish-brown to dark brown, with indistinct purplish suffusion; antemedial and postmedial lines dark and sinuate; subterminal line pale and curved; reniform stigma indistinct. A darker form occurs, in which the fore-wing is almost uniformly dark brown (Fig. 1B). Hind-wing dark brown to blackish, with several separated orange-yellow longitudinal streaks and patches extending from the basal area towards the termen; terminal margin dark. Abdomen yellowish-brown with dark dorsal scaling.

**Figure 2.**
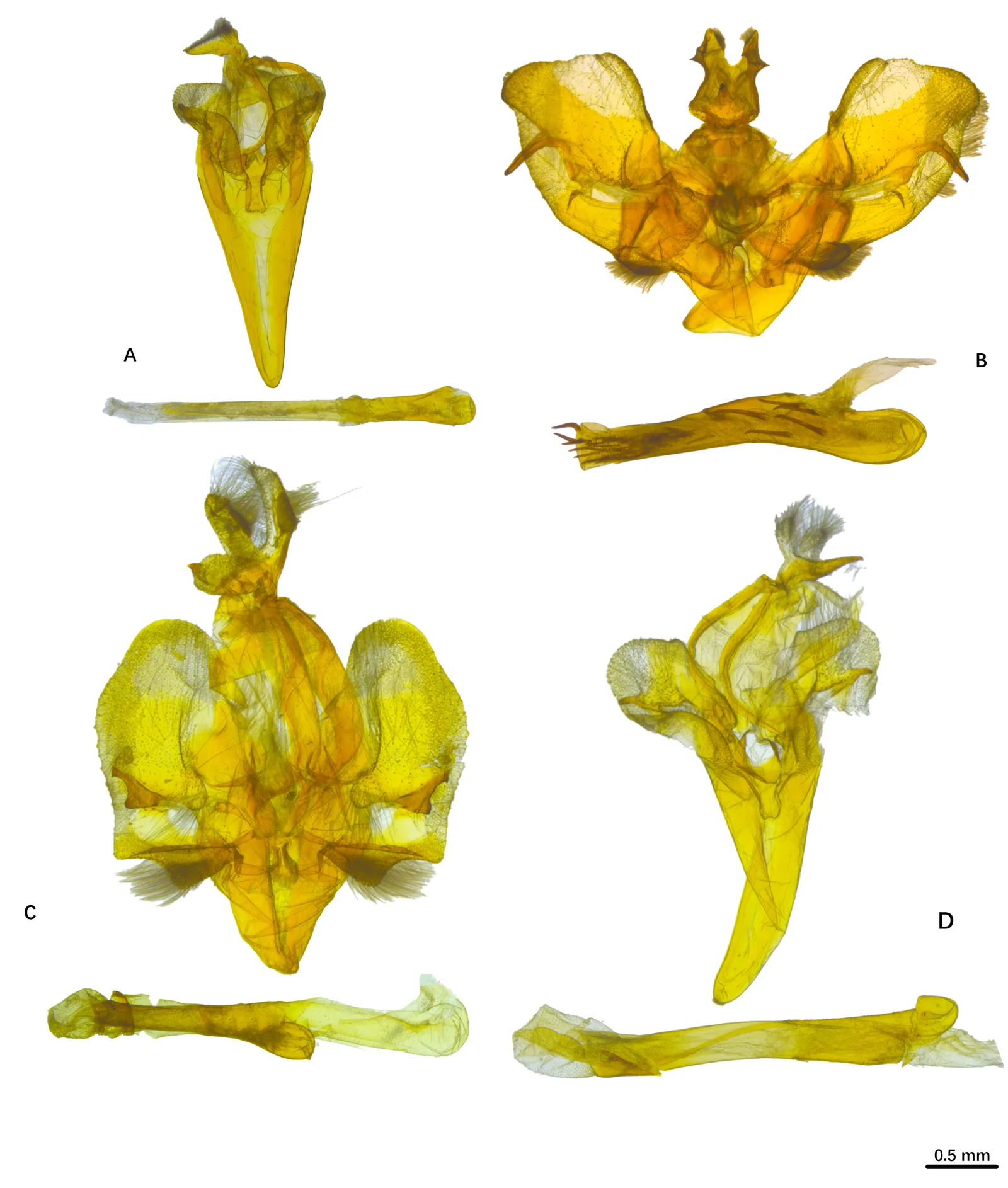
Male genitalia of Chinese *Hypocala* species; **A** *Hypocala subsatura*, slide no. LepiEreb0077; **B** *H. violacea*, slide no. LepiEreb0078; **C** *H. deflorata*, slide no. LepiEreb0079; **D** *H. rostrata*, slide no. LepiEreb0082.

**Male genitalia (Fig. 2A)**. Tegumen narrow and elongate. Uncus short, flattened and blade-like, apically expanded and densely setose. Valvae elongate, somewhat rectangular, weakly tapering to a rounded apex, the apical part finely spinulose, with a small sclerotised process at the base of the sacculus. Vinculum V-shaped, prolonged into a remarkably long, narrowly triangular sacculus that tapers gradually to a rounded apex and distinctly exceeds the valva in length. Aedeagus very long, slender, cylindrical and almost straight, slightly widened and more strongly sclerotised basally; vesica without distinct cornuti, only faint, minutely sclerotised patches in the median part.

**Figure 3.**
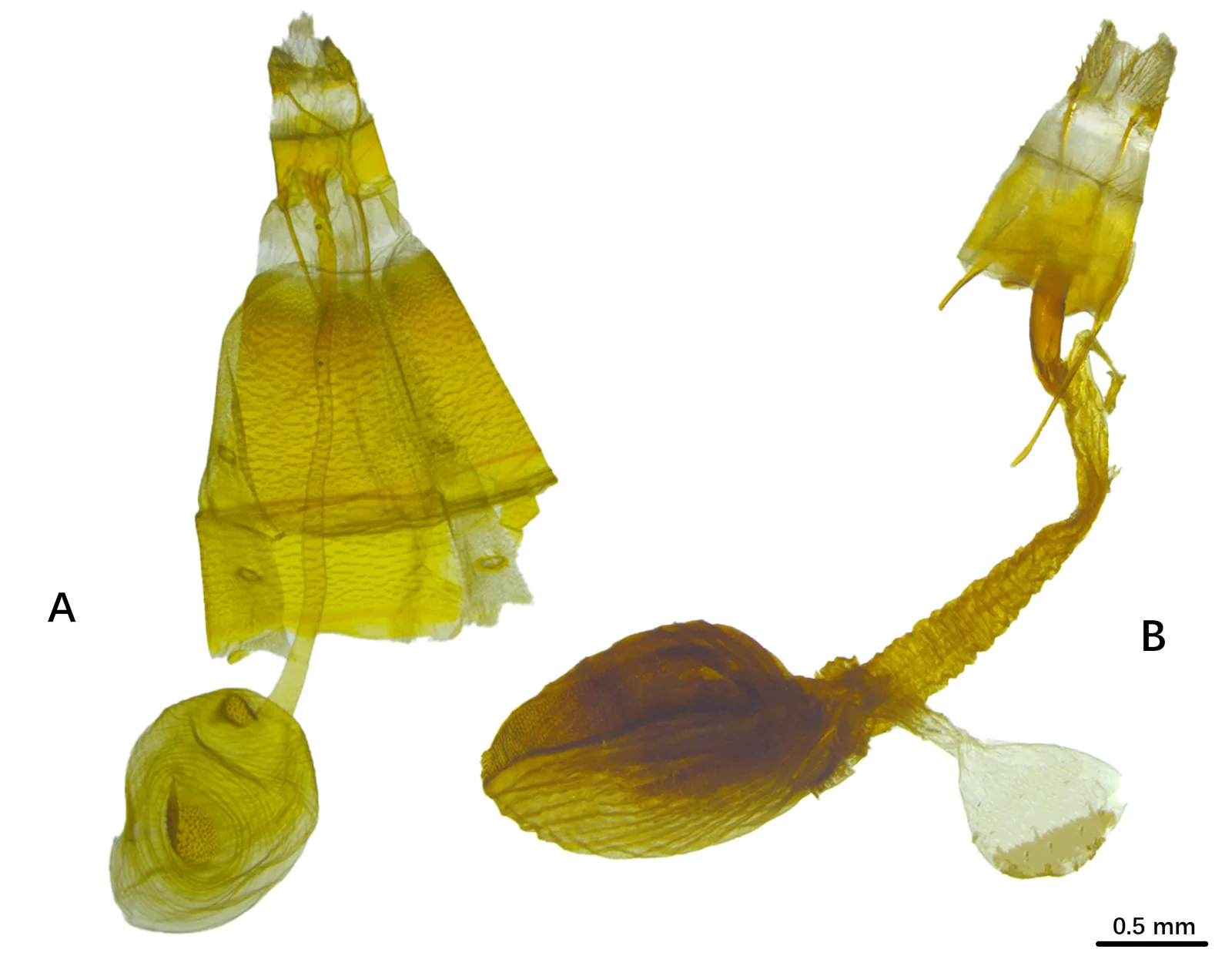
Female genitalia of Chinese *Hypocala* species; **A** *Hypocala subsatura*, slide no. LepiEreb0080; **B** *H. deflorata*, slide no. LepiEreb0081.

**Female genitalia (Fig. 3A)**. Papillae anales broad, subtriangular, densely setose. Apophyses posteriores long and slender; apophyses anteriores about half as long as apophyses posteriores. Abdominal segment VIII broad, trapezoidal, moderately sclerotised, its surface finely reticulate. Ductus bursae long and narrow, membranous, straight, entering the corpus bursae close to its margin. Corpus bursae large, rounded, thin-walled and membranous, finely concentrically striate; two signa present, each composed of minute denticles: a small, rounded one near the entrance of the ductus bursae and a larger, elongate-ovate one on the lateral wall of the corpus bursae.

#### Diagnosis

##### Host Plants

Apple (*Malus* spp.), pear (*Pyrus* spp.), plum (*Prunus* spp.), persimmon (*Diospyros* spp.) and oak (*Quercus* spp.) (Chen 1999, Li 2023).

##### Remarks

This species is polymorphic in fore-wing pattern: the fore-wing is either patterned with a large, twisted, angular dark brown patch bordered by pale grey areas on the costa and dorsum (Fig. 1A-C) or almost uniformly dark reddish-brown to greyish-brown without pale areas (Fig. 1D). It differs from *H. deflorata* in having the hind-wing blackish-brown with separate yellow streaks and spots (Fig. 1A-D) rather than yellow with a black discal spot and a broad black outer band (Fig. 1E and F) and in the very long sacculus of the male genitalia and the two signa of the bursa copulatrix in female genitalia (Fig. 2A and Fig. 3A).

#### Distribution

China (Inner Mongolia, Liaoning, Hebei, Shandong, Henan, Shaanxi, Gansu, Jiangsu, Zhejiang, Fujian, Taiwan, Guangdong, Hainan, Hong Kong, Macao,Yunnan, Xizang); Japan, India, Bangladesh.

### Hypocala violacea

Butler, 1879

22026D43-7BA6-580A-A695-0D982DE95103

Hypocala violacea Butler (1879): 6. Type locality: India.
Hypocala clarissima Butler (1892): 21.
Hypocala kebeae Bethune (1906): 249.
Hypocala violacea Hampson (1894): 454; Holloway (1976): 35; Sugi (1982): 863.

#### Materials

- **Type status:** Other material. **Occurrence:** occurrenceID: 5C702D65-A32C-54FA-AD00-E5ED988DEB45; **Material Entity:** materialEntityID: NWAFU:Ento:LepiEreb0078; materialEntityType: Pinned specimen; discipline: Entomology; preparations: pinned/ genitalia ♂; disposition: in collection; verbatimLabel: CHINA: Hainan, Jianfengling, 980 m, 7.v.2008, collected at light trap, Fu Qiang

#### Description

**Re-description**

**Adult (Fig. 1H)**. Wingspan 50 mm. Head, thorax and fore-wing reddish-brown with a distinct violaceous or purplish suffusion. Fore-wing elongate, with dark antemedial and postmedial lines; subterminal line pale and sinuate; terminal area slightly darker. Hind-wing predominantly dark brown, with yellow markings occupying the basal and median areas; terminal black band relatively narrow. Underside yellow with contrasting dark bands and discal markings. Abdomen yellowish with dark dorsal scaling.

**Male genitalia (Fig. 2B)**. Genital capsule broad. Tegumen broad and hood-like. Uncus broad basally and deeply divided apically into two erect, subparallel lobes; each lobe bears a distinct lateral spur and terminates in a blunt, densely setose apex. Valvae broad and deep, approximately subrectangular, with rounded distal margins; dorsodistal surface finely spinulose. Each valva bears a long, slender, sharply pointed, curved sclerotised process arising medially and directed outwards. Ventral margin with several short, stout, curved spines; ventral angle with a dark sclerotised lobe bearing a dense tuft of long setae. Saccus short, broad and triangular. Aedeagus long and robust, gently curved and basally enlarged. Vesica with several elongate, blade-like cornuti along the median portion and a distal group of slender, needle-like cornuti, some appearing hooked; a thin membranous lobe arises near the basal portion of the vesica.

##### Host Plant

*Diospyros* (Ebenaceae) Holloway (2005).

#### Distribution

China (Taiwan, Hainan, Macao, Yunnan); India, Papua New Guinea.

##### Remarks

The Hainan specimen (Fig. 1H) exhibits a more heavily patterned fore-wing than the typical form described by Holloway (2005) from Borneo. However, the male genitalia (Fig. 2B) agree perfectly with the diagnostic illustrations of *H. violacea* provided by Holloway (2005), particularly in the shape of the uncus, the presence of the long, curved valval processes and the short triangular sacculus. The species has previously been recorded from Macao (Li 2023; Xian 2024), Taiwan and Yunnan (Chen 1999). The Hainan specimen examined here extends the known distribution of the species within China, but further material is needed to confirm its establishment in Hainan.

### Hypocala deflorata

(Fabricius, 1794)

C4D39A75-BCCF-5829-A3AC-CF9874822712

Hyblaea deflorata Fabricius (1794): 127. Type locality: India Orientali.
Hypocala moorei Butler (1892): 21.
Hypocala deflorata Holloway (1976): 36; Chen (1982): 1184; Holloway (2005): 168.

#### Materials

- **Type status:** Other material. **Occurrence:** occurrenceID: 763250E8-95A0-5DD1-BEBC-3FE7558363C6; **Material Entity:** materialEntityType: pinned specimen; discipline: Entomology; preparations: pinned ♂; disposition: in collection; verbatimLabel: CHINA: Yunnan, Dali, Diancang Mountain, 2990 m, 20.vi.1974, Zhou Yao & Yuan Feng
- **Type status:** Other material. **Occurrence:** occurrenceID: 66303FB0-D02B-527B-A09C-8478F0444CD1; **Material Entity:** materialEntityID: NWAFU:Ento:LepiEreb0079; materialEntityType: pinned specimen; discipline: Entomology; preparations: pinned ♂; disposition: in collection; verbatimLabel: CHINA: Shaanxi, Fengxian, 6.ix.1991, Wang Min
- **Type status:** Other material. **Occurrence:** occurrenceID: D0598EEC-337B-5782-AC29-FCAE8D56112A; **Material Entity:** materialEntityID: NWAFU:Ento:LepiEreb0081; materialEntityType: pinned specimen; discipline: Entomology; preparations: pinned ♀; disposition: in collection; verbatimLabel: CHINA: Shaanxi, Yangling, 6.vii.1989, Yuan Feng

#### Description

**Re-description**

**Adult (Fig. 1 E** and **F)**. Wingspan 43–50 mm. Head, thorax and fore-wing pale greyish-brown to violaceous-grey. Fore-wing elongate, with distinct dark antemedial and postmedial lines; subterminal line pale and curved; tornal area slightly paler. Hind-wing bright yellow with a broad black terminal band; inner margin of the black band evenly curved. A narrow yellow mark is present within the black border near the termen. Underside yellow with reduced dark markings. Abdomen yellow with dark transverse bands.

**Male genitalia (Fig. 2C)**. Genital capsule broad. Tegumen broad. Uncus broad basally, its distal portion expanded into a broad, deeply divided crown, densely fringed with setae; each lobe bears a darker sclerotised central area. Valvae broad and rounded, with the distal inner surface finely granulose and the outer and ventral margins densely setose; a dense tuft of long setae is present at the dorsodistal angle and a small triangular sclerotised process is present on the inner surface of the ventral portion. Saccus short, triangular, with a pointed apex. Aedeagus long and moderately stout, with a rounded, strongly sclerotised basal coecum. Vesica large and membranous, approximately as long as the aedeagus shaft and strongly recurved distally; no conspicuous group of large cornuti is evident, although fine sclerotised granules present.

**Female genitalia (Fig. 3B)**. Papillae anales narrow and elongate, apically pointed, sparsely setose. Apophyses posteriores very long and slender, distinctly longer than in *H. subsatura*. Abdominal segment VIII short, subquadrate, weakly sclerotised. Ductus bursae long, sinuous, broader than in *H. subsatura*, sclerotised and transversely corrugated, with a dark, heavily sclerotised, cylindrical antrum. Corpus bursae ovate, dark brown, heavily sclerotised over most of its surface, granulose distally, with longitudinal striae on one side; a large, pale, membranous, pear-shaped appendix bursae at the junction with the ductus bursae; signum not apparent.

##### Host Plant

Persimmon (*Diospyros* spp.) (Chen 1999).

#### Distribution

China (Hebei, Shandong, Shaanxi, Jiangsu, Zhejiang, Jiangxi, Fujian, Taiwan, Guangdong, Hainan, Hong Kong, Macao, Sichuan, Guizhou, Yunnan); Russia, Japan, North Korea, South Korea, Vietnam, Thailand, India, Nepal, Sri Lanka, Indonesia, USA (Hawaii), Africa.

### Hypocala rostrata

(Fabricius, 1794)

9CC05A51-4072-51B0-A0CB-D56A3ED38CDE

Hyblaea rostrata Fabricius (1794): 127. Type locality: India Orientali [East Indies].
Hypocala plumicornis Guenée (1852): 75.
Hypocala angulipalpis Guenée (1852): 77.
Hypocala efflorescens Guenée (1852): 77.
Hyblaea genuina Wallengren (1856): 66.
Hyblaea bohemani Wallengren (1856): 67.
Hypocala rostrata Chen (1982): 1184; Berio (1955) & Poole (1989): 539.

#### Materials

- **Type status:** Other material. **Occurrence:** occurrenceID: 7AB32DBB-4184-5F4D-8ADD-4F3947B52E73; **Material Entity:** materialEntityID: NWAFU:Ento:LepiEreb0082; materialEntityType: pinned specimen; discipline: Entomology; preparations: pinned/ genitalia **♂**; disposition: in collection; verbatimLabel: CHINA: Sichuan; materialEntityRemarks: collector and date information missing; provenance from Sichuan is consistent with the historical record.

#### Description

**Re-description**

**Adult (Fig. 1G)**. Wingspan 36 mm. Head and thorax pale greyish-brown. Fore-wing light greyish-brown, with indistinct transverse lines and weak purplish suffusion; subterminal line pale and curved. Hind-wing bright yellow with a broad black terminal band; inner margin of the black band irregular and forming a distinct inward projection near the middle of the wing. Underside yellow with dark bands and discal marks. Abdomen yellowish with dark dorsal scaling.

**Male genitalia (Fig. 2D)**. Tegumen broad and hood-like. Uncus narrow, deeply U-notched apically to form two divergent lobes, each with a small lateral spur and a hooked tip. Valvae broad and deep, approximately rectangular, with rounded distal margins; dorsodistal part finely spinulose; each valva bearing a long, slender, sharply pointed, curved sclerotised process arising medially and directed towards the outer margin; ventral margin with a few short, stout, curved spines; ventral angle with a dark sclerotised lobe bearing a dense tuft of long setae. Saccus short, broad and triangular. Aedeagus long and robust, gently curved, with a bulbous base. Vesica with a single conspicuous, sharply pointed cornutus; no distal cluster of slender cornuti evident in the examined specimen. A thin membranous lobe arises near the basal portion of the vesica.

##### Host Plants

Four Ebenaceae species (*Diospyros abyssinica*, *Diospyros kaki*, *Diospyros melanoxylon* and an unidentified *Royena* sp.), *Pouteria* sp. (Sapotaceae) and *Citrus* sp. (Rutaceae) (DePrins and DePrins 2022).

#### Distribution

China (Taiwan, Hong Kong, Sichuan); India.

##### Remarks

This species is most closely related to *H. deflorata*, from which it can be distinguished by the following characters: fore-wing light greyish-brown with indistinct transverse lines (versus purplish-grey with distinct lines in *H. deflorata*); hind-wing with a broad black terminal band bearing a distinct tooth-like projection inwards at the middle of the inner margin (versus a broad band without a tooth-like projection in *H. deflorata*). The male genitalia of *H. rostrata* are similar to those of *H. violacea* in the general configuration of the valvae and long curved valval processes, but differ notably in the shape of the uncus and in the vesical armature. In *H. rostrata*, the uncus forms two more divergent lobes with hooked apices and the vesica bears a single conspicuous, sharply pointed cornutus, whereas *H. violacea* has a more complex vesical armature without cornuti (Fig. 2B and D).

### Hypocala andamana

Wileman, 1923

DCB0062D-A1B7-5C7A-A5A4-ED1EE56FDD9D

Hypocala andamana Wileman (1923): 280. Type locality: Andaman Islands.
Hypocala lativitta Moore (1877): 606 (junior primary homonym of *Hypocala lativitta* Walker (1865)).

#### Description

**Re-description**

We did not examine specimens of this species. The following re-description is based entirely on literature (Holloway 2005). *H. andamana* is distinguished from its congeners by the extensively yellow basal area of the hind-wing and the pale grey fore-wing with black and buff distal patches (Holloway 2005).

#### Distribution

China (Taiwan); India (Meghalaya, Nagaland, Chhattisgarh, Tamil Nadu, Andaman and Nicobar Islands), Thailand, Sundaland, Sulawesi.

##### Remarks

*Hypocala andamana* is recorded here as part of the Chinese fauna, based solely on historical literature records from Taiwan (Holloway 2005; Taiwan Biodiversity Network). No specimens of this species were available in the NWAFU collection or on loan during this study. The species was originally described as *Hypocala lativitta*, which is a junior primary homonym of *Hypocala lativitta* Walker (1865) and was subsequently replaced by *H. andamana*. It can be distinguished from the other Chinese *Hypocala* species by the extensively yellow basal area of the hind-wing and the pale grey fore-wing with distinct black and buff distal patches (Holloway 2005). As no material was available for dissection, the male and female genitalia of this species are not illustrated or described in the present study. Future collections from Taiwan and adjacent regions are needed to confirm its morphological variability and to provide a detailed re-description of the genitalia.

## Identification Keys

### Key to the Chinese species of *Hypocala* Guenée, 1852

**Table:** 

1	Hind-wing predominantly blackish-brown, with several separate orange-yellow longitudinal streaks and spots; without a continuous broad black terminal band.	*H. subsatura*
–	Hind-wing predominantly yellow or dark brown with yellow markings, with a distinct black terminal band.	2
2	Hind-wing with the basal area extensively yellow; fore-wing pale grey with black and buff distal patches (not examined in this study; recorded from Taiwan).	*H. andamana*
–	Hind-wing with yellow markings more restricted or, if extensive, then fore-wing reddish-brown or greyish-brown with distinct transverse lines.	3
3	Hind-wing dark brown with yellow markings, black terminal band relatively narrow; fore-wing reddish-brown with a distinct purple tinge.	*H. violacea*
–	Hind-wing yellow with a broad black terminal band; fore-wing greyish-brown to purplish-grey.	4
4	Fore-wing purplish-grey to greyish-brown, with distinct dark transverse lines; inner margin of black terminal band on hind-wing without a distinct tooth-like projection.	*H. deflorata*
–	Fore-wing light greyish-brown, with transverse lines indistinct; inner margin of black terminal band on hind-wing with a distinct tooth-like projection directed inwards near the middle.	*H. rostrata*

## Supplementary Material

XML Treatment for Hypocala

XML Treatment for Hypocala subsatura

XML Treatment for Hypocala violacea

XML Treatment for Hypocala deflorata

XML Treatment for Hypocala rostrata

XML Treatment for Hypocala andamana
